# Efficacy and safety of maintenance immune checkpoint inhibitors with or without pemetrexed in advanced non-squamous non-small cell lung cancer: a retrospective study

**DOI:** 10.1186/s12885-022-09674-2

**Published:** 2022-05-24

**Authors:** Xiaodong Gu, Zhiyong Shi, Lan Shao, Yuxin Zhang, Yiping Zhang, Zhengbo Song, Wenxian Wang, Guangyuan Lou

**Affiliations:** 1 The Second Clinical Medical College of Zhejiang Chinese Medical University, Hangzhou, 310053 Zhejiang China; 2 Department of Thoracic Medical Oncology, Chinese Academy of Sciences University Cancer Hospital (Zhejiang Cancer Hospital), No.1 Banshan East Street, Gongshu District, Hangzhou, 310022 China; 3 Institute of Basic Medicine and Cancer (IBMC), Chinese Academy of Sciences, Hangzhou, 310022 Zhejiang China; 4 Department of Radiotherapy, Hangzhou Cancer Hospital, Hangzhou, 310002 Zhejiang China

**Keywords:** Maintenance therapy, Pemetrexed, Immune checkpoint inhibitors, Non-squamous non-small cell lung cancer, Adverse events

## Abstract

**Background:**

Advanced non-squamous non-small cell lung cancer (NS-NSCLC) patients without driver gene mutations are usually treated with immune checkpoint inhibitors (ICIs) plus pemetrexed as maintenance therapy after first-line ICIs plus 4–6 cycles of pemetrexed/platinum. Some patients in the real world receive ICIs monotherapy as maintenance therapy. No clinical study has compared the efficacy and safety of ICIs with or without pemetrexed as maintenance therapy.

**Methods:**

We performed a retrospective study analyzing clinical data of patients with NS-NSCLC who were diagnosed in Zhejiang Cancer Hospital from September 2018 to May 2021 and received maintenance therapy after 4–6 cycles of ICIs plus pemetrexed/platinum. Patients were divided into ICIs plus pemetrexed group and ICIs monotherapy group. Progression Free Survival 1 (PFS1) and PFS2, defined as the interval from the date of initial treatment and maintenance therapy to the date of systemic progression/death or the last follow-up, respectively.

**Results:**

A total of 120 patients received ICIs with or without pemetrexed as maintenance therapy. Eighty-two patients received ICIs plus pemetrexed as maintenance therapy, and 38 patients received ICIs monotherapy. There were no statistically significant difference in median PFS1 between the ICIs monotherapy group and ICIs plus pemetrexed group (12.00 months vs. 12.07 months, *P* = 0.979). Among patients with PD-L1 TPS < 1%, the median PFS1 was worse with ICIs monotherapy (9.50 months vs. 14.20 months, *P* = 0.039). Among patients with PD-L1 TPS ≥50% or 1–49%, the median PFS1 in both groups was not statistically significant (*P* = 0.866, *P* = 0.589, respectively). Results for median PFS2 were similar to median PFS1, with statistically significantly different only in patients with PD-L1 TPS < 1% (*P* = 0.008). The 2-year survival rates of the two groups were similar (66.7% vs. 69.5%, *P* = 0.812). The incidence of fatigue was significantly higher in the ICIs plus pemetrexed group (*P* = 0.023).

**Conclusions:**

ICIs with or without pemetrexed can be used as maintenance therapy after first-line ICIs plus 4–6 cycles of pemetrexed/platinum in patients with advanced NS-NSCLC based on PD-L1 expression.

## Background

The incidence and mortality of lung cancer are still among the top malignant tumors globally [1]. Non-small cell lung cancer (NSCLC) accounts for 85%, with non-squamous tissue type as the primary subtype [2]. The revolutionary development of immune checkpoint inhibitors (ICIs) has significantly changed the treatment model for lung cancer in recent years. Based on multiple studies, first-line ICIs combined with chemotherapy significantly prolonged the progression-free survival (PFS) and overall survival (OS) of patients with advanced non-squamous NSCLC (NS-NSCLC) and has become the first-line standard treatment for NSCLC patients without driver gene mutations [3–6]. Therefore, the efforts to improve the treatment effect are mainly reflected in the combination of new ICIs or the improvement of maintenance treatment programs.

Maintenance therapy in the National Comprehensive Cancer Network (NCCN) guidelines refers to using at least one of the agents given in the first line, beyond 4–6 cycles, in the absence of disease progression [7]. In patients with NS-NSCLC, the PARAMOUNT study showed that pemetrexed as continuous maintenance therapy could reduce the risk of disease progression and prolong progression-free survival [8, 9]. Based on the AVAPERL and COMPASS studies, bevacizumab plus pemetrexed as maintenance therapy is recommended after first-line bevacizumab plus pemetrexed/platinum [10, 11]. The success of immune checkpoint inhibitors heralds the dawn of a new age in maintenance therapy. Studies such as KEYNOTE189, CameL, and RATIONALE304 usually design ICIs plus pemetrexed as maintenance therapy after ICIs plus 4–6 cycles of pemetrexed/platinum. However, there are still a few patients in the real world do not receive pemetrexed for some reason and choose ICIs monotherapy as maintenance therapy. In addition, a minority of patients with ICIs plus pemetrexed maintenance discontinue pemetrexed after maintenance for fewer cycles. In patients with PD-L1 TPS ≥1% or ≥ 50%, the “chemo-free” mode of pembrolizumab monotherapy still improved overall survival and avoided chemotherapy-induced adverse events [12, 13]. The results imply that chemotherapy may not be required for maintenance therapy in these patients.

Importantly, no clinical studies have investigated the difference in the efficacy and safety of ICIs with or without pemetrexed as first-line maintenance therapy in NS-NSCLC. We conducted a retrospective study to investigate whether ICIs combined with pemetrexed as maintenance therapy have clinical benefits in patients with advanced NS-NSCLC. We also stratified patients for their PD-L1 expression to achieve precise benefit.

## Methods

### Patients

All patients were diagnosed with NS-NSCLC in Zhejiang Cancer Hospital from September 2018 to May 2021. Inclusion criteria: 1) First-line use of ICIs plus with pemetrexed/platinum (cisplatin or carboplatin) for 4–6 cycles; 2) Complete response, partial remission or stable disease after 4–6 cycles of induction chemotherapy; 3) Eastern Cooperative Oncology Group Performance Status (ECOG PS) 0–1; 4) Complete baseline clinical data, including diagnostic age, gender, smoking, ECOG PS, intrathoracic metastasis status, liver metastasis status, bone metastasis status, brain metastasis status, programmed cell death-ligand 1 (PD-L1) expression and Adverse events. Exclusion criteria: 1) Pathologically diagnosed as squamous cell carcinoma; 2) EGFR, ALK, and ROS1 genes are positive; 3) ECOG PS 2–4. This study was approved by the Ethics Committee of the University Cancer Hospital of the Chinese Academy of Sciences (Zhejiang Cancer Hospital). It was carried out under the Declaration of Helsinki.

### Study design and treatment

Patients received 4–6 cycles of ICIs plus pemetrexed/platinum (cisplatin or carboplatin) in the induction phase. Patients who achieved complete remission (CR), partial remission (PR), or stable disease (SD) after induction entered the maintenance period with the patient’s consent. Under normal circumstances, the attending physician would recommend pemetrexed combined with ICIs as maintenance therapy. If the patient refused to continue maintenance of pemetrexed or is intolerant to chemotherapy, ICIs monotherapy was used for maintenance therapy. According to the maintenance regimen, patients were divided into ICIs plus the pemetrexed group and ICIs monotherapy group. The type of ICIs used for NS-NSCLC was predominantly pembrolizumab (37.5%), with the remaining therapy including camrelizumab (23.3%), sintilimab (21.7%), tislelizumab (10.0%) and toripalimab (7.5%). All chemotherapy and ICIs were performed under the standard doses of NCCN guidelines. PD-L1 expression was evaluated using PD-L1 22C3 pharmDx (Agilent Technologies, Santa Clara, CA, USA). We used the Response Evaluation Criteria in Solid Tumors (RECIST) v1.1 to assess the response to each treatment. Before the analysis, two oncologists checked the efficacy. They assessed the tumor response according to RECIST v1.1 through chest computed tomography and/or brain magnetic resonance imaging (MRI) every 4–8 weeks. The objective response rate (ORR) is defined as the proportion of patients with CR plus PR. Progression-free survival 1 (PFS1) was defined as the interval from the date of initial treatment to the date of systemic progression/death or the last follow-up, whichever is the first to trigger the review of the event date. Progression-free survival 2 (PFS2) was defined as interval from the date of maintenance therapy to the date of systemic progression/death or the last follow-up. Overall survival (OS) was defined as the interval from the date of diagnosis of advanced NS-NSCLC to the date of death or the last follow-up. Safety assessments included physical examination, documentation of adverse events, electrocardiogram, and laboratory tests. Adverse events were graded according to National Cancer Institute Common Terminology Criteria for Adverse Events (CTCAE) version 5.0.

### Statistical analysis

Chi-square test or Fisher’s exact test were used to test the association between the treatment group of all patients and the clinical classification variables. Kaplan-Meier curves were used for univariate survival analysis. The Cox proportional hazards model was used to complete the univariate and multivariate survival analyses with a hazard ratio (HR) and corresponding 95% confidence interval (95%CI). Significance between groups was defined as *p* values < 0.05. SPSS 26.0 (IBM) and GraphPad Prism version 9.0 (GraphPad Software) were used for statistical analysis of data.

## Results

### Patient characteristics

**Fig. 1:**
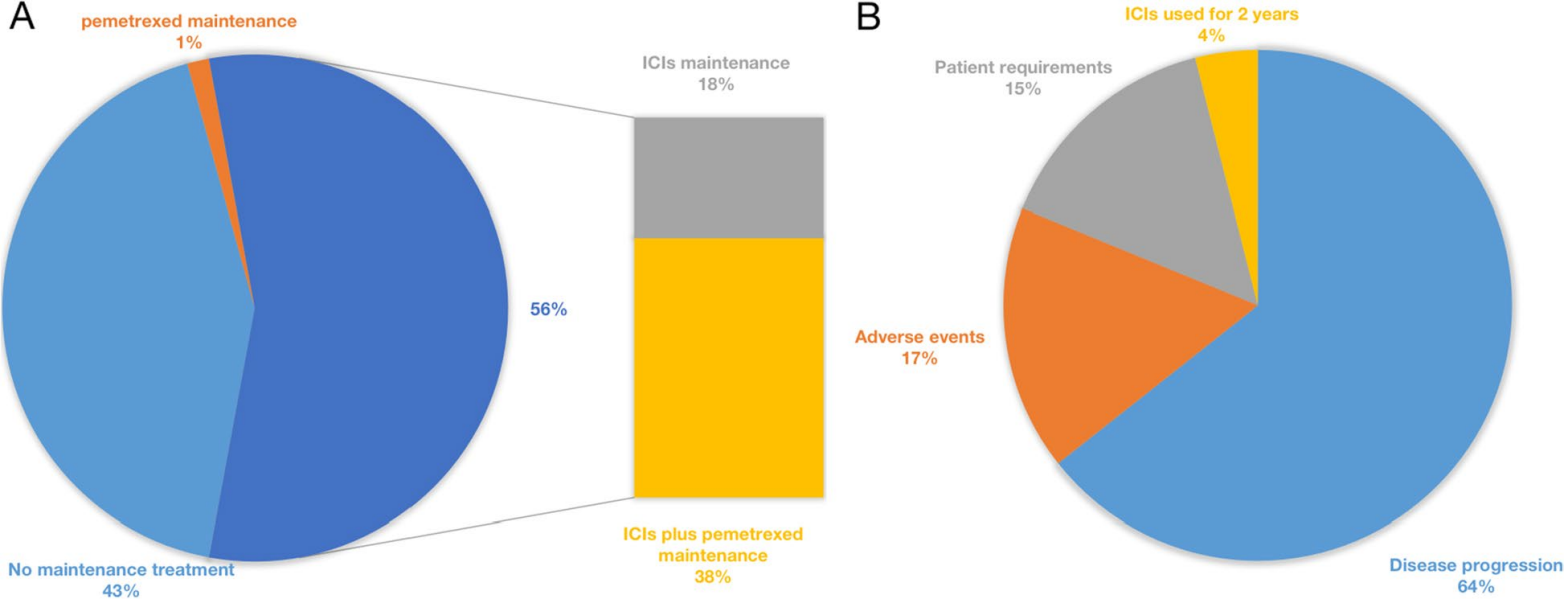
**A** Maintenance treatment options after 4–6 cycles of induction therapy in 215 patients. **B** Reasons for maintenance treatment discontinuation

From September 2018 to May 2021, 215 patients received first-line ICIs plus pemetrexed/platinum (cisplatin or carboplatin) at Zhejiang Cancer Hospital, and 123 (57.2%) patients entered the maintenance phase. Among them, three patients (1.4%) received pemetrexed as maintenance therapy, 38 patients (17.7%) received ICIs monotherapy as maintenance therapy, and 82 patients (38.1%) received ICIs plus pemetrexed as maintenance therapy (Fig. 1A). Of the 38 patients, 23 (60.5%) did not receive pemetrexed as maintenance therapy due to adverse events such as myelosuppression, and 15 (39.5%) were due to patients’ refusal. Among 82 patients, 25 patients (30.4%) discontinued pemetrexed during maintenance therapy due to adverse events such as fatigue or myelosuppression. The 120 patients who received ICIs with or without pemetrexed as maintenance therapy were included in this study. Among 120 patients, 101 patients stopped maintenance therapy, 65 patients (64.4%) stopped due to disease progression, 17 patients (16.8%) due to adverse events, 15 patients (14.9%) requested to stop maintenance therapy (including economic reasons, unwillingness to continue, etc.). Four patients (4.0%) stopped maintenance treatment due to ICIs for two years (Fig. 1B). There was no statistical difference in baseline characteristics between the groups (Table 1).

**Table 1:** Baseline patient characteristics at the start of maintenance therapy

Characteristic	All (***n*** = 120)	ICIs (***n*** = 38)	ICIs + Pem (***n*** = 82)	***P***
Age				
≥ 65	53 (44.2)	20 (52.6)	33 (40.2)	0.204
< 65	67 (55.8)	18 (47.4)	49 (59.8)	
Gender				
Male	85 (70.8)	27 (71.1)	58 (70.7)	0.971
Female	35 (29.2)	11 (28.9)	24 (29.3)	
Smoking				
Yes	75 (62.5)	23 (60.5)	52 (63.4)	0.761
No	45 (37.5)	15 (39.5)	30 (69.3)	
ECOG PS				
0	42 (35.0)	13 (34.2)	29 (35.3)	0.902
1	78 (65.0)	25 (65.8)	53 (64.6)	
Brain metastasis				
Yes	17 (14.2)	3 (7.9)	14 (17.1)	0.180
No	103 (85.8)	35 (92.1)	68 (82.9)	
Liver metastasis				
Yes	11 (9.2)	3 (7.9)	8 (9.8)	1.000
No	109 (90.8)	35 (92.1)	74 (90.2)	
Intrathoracic metastasis				
Yes	79 (65.8)	26 (68.4)	53 (64.6)	0.684
No	41 (34.2)	12 (31.6)	29 (35.4)	
Bone metastasis				
Yes	44 (36.7)	12 (31.6)	32 (39.0)	0.431
No	76 (63.3)	26 (68.4)	50 (61.0)	
Platinum				
Cisplatin	33 (27.5)	10 (26.3)	23 (28.0)	0.843
Carboplatin	87 (72.5)	28 (73.7)	59 (72.0)	
PD-L1 TPS				
< 1%	32 (26.7)	10 (26.3)	22 (26.8)	0.370
1–49%	21 (17.5)	4 (10.5)	17 (20.7)	
≥ 50%	32 (26.7)	12 (31.6)	20 (24.3)	
Unkown	35 (29.2)	12 (31.5)	23 (28.0)	
Response to induction therapy				
Partial response	55 (45.8)	12 (31.6)	43 (52.4)	0.033
Stable disease	65 (54.2)	26 (68.4)	39 (47.6)	
Cycles of induction therapy Median (range)	4 (4–6)	4 (4–6)	4 (4–6)	–

*ICIs* Immune checkpoint inhibitors, *Pem* Pemetrexed, *ECOG PS* Eastern Cooperative Oncology Group Performance Status, *PD-L1 TPS* Programmed death ligand 1 tumour proportion score

### Efficacy

In the ICIs monotherapy group, 12 patients achieved PR after induction therapy, with an ORR of 31.6%. In the ICIs plus pemetrexed group, 43 patients achieved PR after induction chemotherapy, with an ORR of 52.4%. The median number of cycles was 8 (range, 2 to 48 cycles) in the ICIs monotherapy group. The median number of ICIs maintenance cycles was 9 (range, 2 to 49 cycles) and pemetrexed maintenance cycles was 6 (range, 2 to 49 cycles) in the ICIs plus pemetrexed group.

**Fig. 2:**
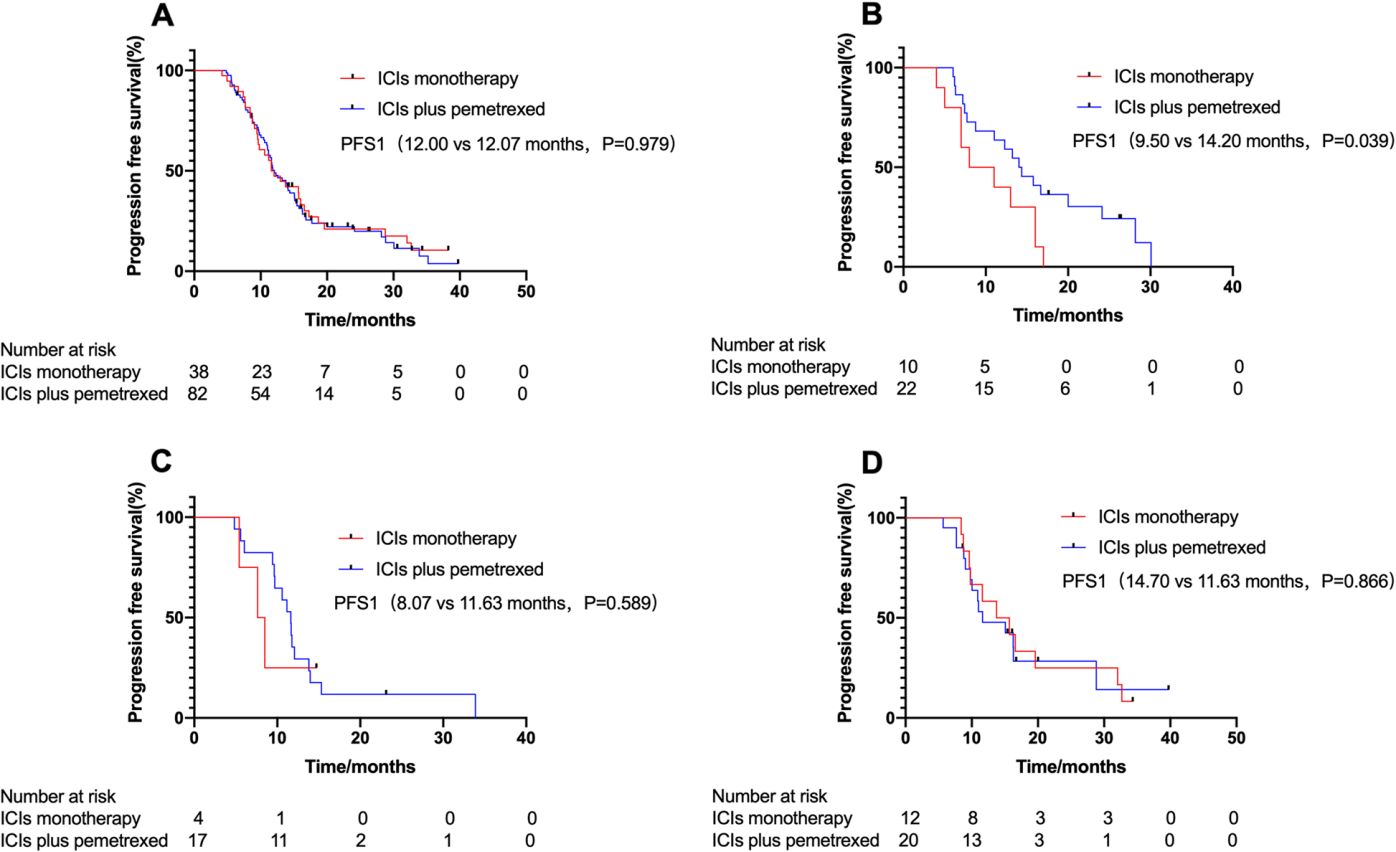
Kaplan-Meier estimates of progression-free survival 1 (PFS1). **A** PFS1 in 120 patients. **B** PFS1 in patients with PD-L1 TPS < 1%. **C** PFS1 in patients with PD-L1 TPS 1–49%. **D** PFS1 in patients with PD-L1 TPS ≥50%

There were no statistically significant differences in median PFS1 between the ICIs monotherapy group and ICIs plus pemetrexed group (12.00 months vs. 12.07 months, HR, 0.95, *P* = 0.979) (Fig. 2A). In the ICIs plus pemetrexed group, 25 patients discontinued pemetrexed during maintenance therapy due to intolerance, and 57 patients did not discontinue pemetrexed. However, whether pemetrexed was terminated or not did not affect the final efficacy (15.07 months vs. 11.67 months, HR, 0.63, *P* = 0.069).

Concerning PD-L1 expression, among patients with PD-L1 TPS < 1%, the median PFS1 in the ICIs monotherapy group was significantly lower than that in the ICIs plus pemetrexed group (9.50 months vs. 14.20 months, HR, 2.15, *P* = 0.039) (Fig. 2B). Among patients with PD-L1 TPS ≥50% or 1–49%, the median PFS1 in both groups was not statistically significant (HR, 0.94, *P* = 0.866; HR, 1.40, *P* = 0.589, respectively) (Fig. 2C-D).

**Fig. 3:**
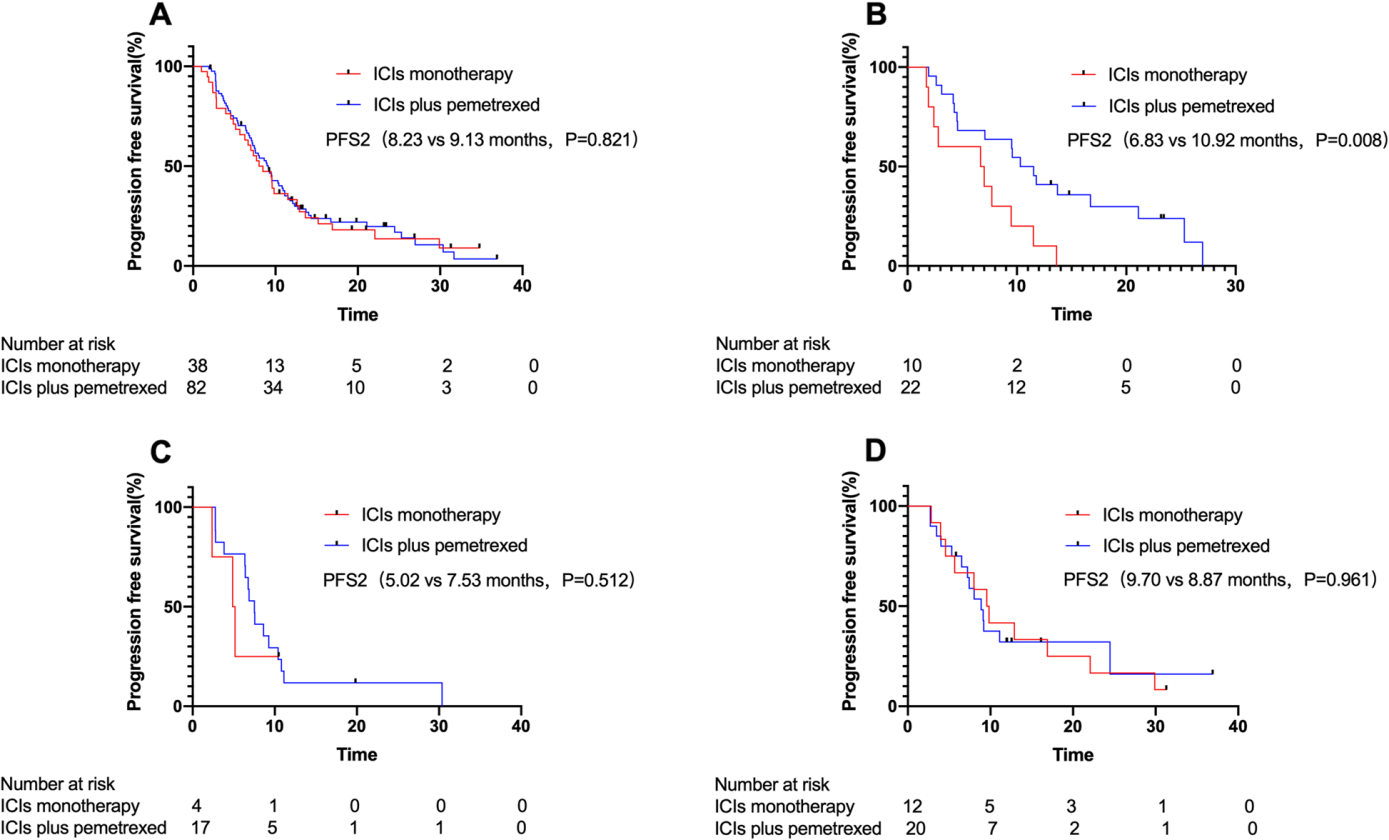
Kaplan-Meier estimates of progression-free survival 2 (PFS2). **A** PFS2 in 120 patients. **B** PFS2 in patients with PD-L1 TPS < 1%. **C** PFS2 in patients with PD-L1 TPS 1–49%. **D** PFS2 in patients with PD-L1 TPS ≥50%

Results for median PFS2 were similar to median PFS1, with no statistical difference between ICIs monotherapy group and ICIs plus pemetrexed group (*P* = 0.821) (Fig. 3A). Similarly, the median PFS2 was statistically significantly different only in patients with PD-L1 TPS < 1% (*P* = 0.008) (Fig. 3B-D).

**Fig. 4:**
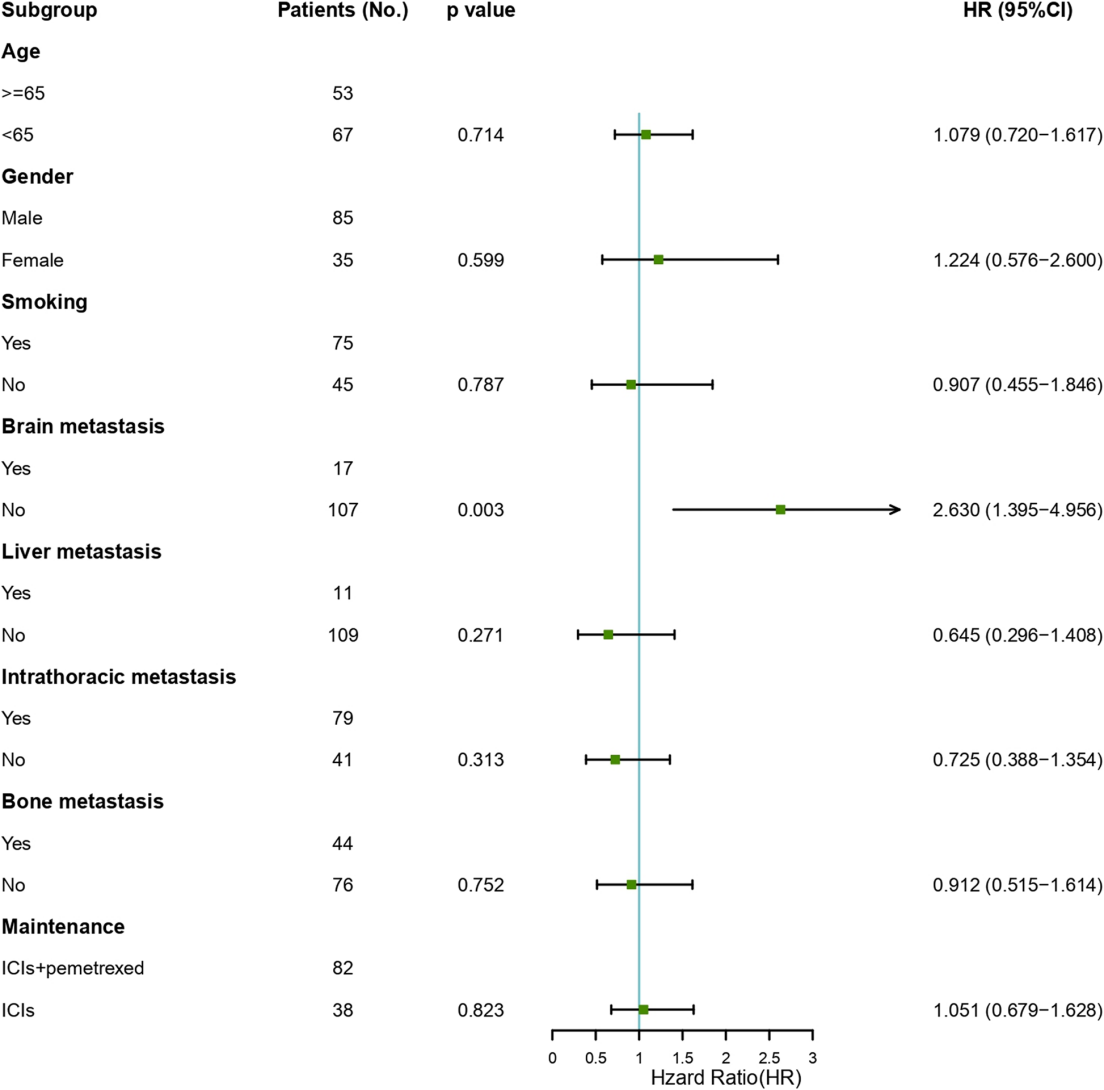
Multivariate analyses of factors associated with Progression-free survival

A subsequent univariate analysis illustrated that the features such as Age, Gender, Smoking, Liver metastasis, Intrathoracic metastasis, Bone metastases, and ICIs plus pemetrexed or ICIs Maintenance were not independent influence factors PFS. The results of the multivariate analysis indicated that brain metastasis was an independent factor affecting PFS (HR = 2.630; 95%CI, 1.395 to 4.956, *P* = 0.003). The detailed data is shown in Fig. 4.

Median follow-up was 23.20 months (95%CI 20.04–26.34) from the first day of diagnosis of advanced NS-NSCLC. As of December 31, 2021, 37 patients (30.8%, 37/120) had died in the two groups, and the 2-year survival rate of the ICIs monotherapy group and ICIs plus pemetrexed group were 66.7 and 69.5%, respectively. The difference was not statistically significant (*P* = 0.812).

### Safety

During the maintenance treatment of 120 patients, 60 patients (60/120, 50.0%) experienced 100 Adverse events (AEs), mostly grade 1 or 2 (79/100, 79.0%). The most common any-grade AEs were hematologic toxicity, pneumonia, and fatigue (Table 2). Importantly, fatigue was more common in the ICIs plus pemetrexed group (22.0% vs. 5.3%, *P* = 0.023). Decreased appetite (7.3% vs. 0.0%, *P* = 0.207), hematologic toxicity (23.2% vs. 18.4%, *P* = 0.557), pneumonia (14.6% versus 7.9%, *P* = 0.458), peripheral edema (6.1% vs. 2.6%, *P* = 0.719) were also more likely to appear in the ICIs plus pemetrexed group, but the difference was not statistically significant. During maintenance, No patients in two groups had grade 5 AEs. 6 patients (15.8%) in the ICIs monotherapy group and 15 patients (18.3%) in the ICIs plus pemetrexed group experienced grade 3–4 AEs (*P* = 0.938).

**Table 2:** Drug-related adverse events

Adverse events	ICIs maintenance			ICIs + Pem maintenance		
	All (***n*** = 24)	Grade 1–2 (***n*** = 18)	Grade 3–4 (***n*** = 6)	All (***n*** = 76)	Grade 1–2 (***n*** = 61)	Grade 3–4 (***n*** = 15)
Hematologic toxicity	7	6	1	19	10	9
Pneumonia	3	2	1	12	7	5
Thyroid/hyperglycemia	2	2	0	6	6	0
Elevated LFTs	2	1	1	2	2	0
Diarrhea or colitis	2	1	1	1	1	0
Fatigue	2	2	0	18	18	0
Skin/rash	5	3	2	3	2	1
Peripheral edema	1	1	0	5	5	0
Decreased appetite	0	0	0	6	6	0
RCCEP	0	0	0	4	4	0

*ICIs* Immune checkpoint inhibitors, *Pem* Pemetrexed, *LFTs* Liver function tests, *RCCEP* Reactive cutaneous capillary endothelial proliferation

## Discussion

To the best of our knowledge, this is the first study to compare the maintenance of ICIs monotherapy or ICIs plus pemetrexed after ICIs plus 4–6 cycles of pemetrexed/platinum in the first line of advanced NSCLC based on PD-L1 expression. In this study, comparing with ICIs plus pemetrexed maintenance, ICIs monotherapy maintenance showed the same efficacy with PD-L1 TPS ≥1%, and the incidence of fatigue in AEs was less. Therefore, we considered ICIs monotherapy maintenance is an effective and safe first-line maintenance method.

According to several prospective studies, first-line ICIs plus pemetrexed/platinum have become the first-line treatment for NS-NSCLC patients without driver gene mutations. These studies were designed with ICIs plus pemetrexed as maintenance therapy, reached the median PFS of 9.0–11.3 months and median OS of nearly two years [3–6]. There was no significant difference in median PFS1 and PFS2 between the ICIs monotherapy maintenance group and the ICIs plus pemetrexed group in our study. And in the ICIs plus pemetrexed maintenance group, there was no significant difference in median PFS1 and PFS2 with or without pemetrexed discontinuation. In addition, similar results were obtained for the 2-year survival rate in the two groups. These results give us an insight that the overall efficacy of ICIs maintenance alone is no worse than that of ICIs plus pemetrexed maintenance.

Notably, the discontinuation rate for pemetrexed was 30.4% in the ICIs plus pemetrexed maintenance group in our study, which was higher than the 5% in the pemetrexed maintenance group in the previous PARAMOUNT study. We consider that the 30.4% discontinuation rate in our study may be due to the patient’s refusal to continue chemotherapy when these patient’s adverse events reached grade 2, and the clinical oncologist was more willing to comply with the patient’s wishes. While this may indeed affect the generalizability of the results, it also reflects the uniqueness of originating from real-world research.

As we all know, the tumor PD-L1 expression level is considered a biomarker associated with the efficacy of ICIs [14–17]. However, it is not known whether the selection of maintenance therapy should be based on baseline PD-L1 expression. A study analyzed PD-L1 TPS (≥ 50% vs. < 50%) in univariate analysis either in the ICIs with or without chemotherapy group was not an influencing factor of PFS (*P* = 0.20, *P* = 0.54, respectively) [18]. In our study, further analysis of PD-L1 expression showed no difference in median PFS1 and PFS2 regardless of whether TPS was ≥50% or 1–49%. It is suggested that maintenance with ICIs monotherapy is sufficient in patients with PD-L1 TPS ≥1%. In addition, the median PFS1 and PFS2 maintained by ICIs monotherapy was significantly lower in patients with TPS < 1% than in the ICIs plus pemetrexed maintenance group, suggesting that maintenance with ICIs monotherapy is not sufficient in PD-L1-negative patients. We considered that PD-L1 expression could be a stratification factor for maintenance therapy patients.

For safety, common adverse events in prospective studies of ICIs plus pemetrexed as maintenance therapy were hematologic toxicity, pneumonitis, hepatic function abnormality, and fatigue [3–6]. The incidence of fatigue in these studies was around 30–40%. In our study, the incidence of fatigue was significantly lower in the ICIs group than in the ICIs plus pemetrexed group. In addition, there was no significant difference in the incidence of grade 3–4 AEs between the two groups. The safety of ICIs monotherapy maintenance was superior to ICIs plus pemetrexed maintenance.

The relationship between chemotherapy and immunotherapy has not been fully explored. Chemotherapy acts synergistically with immunotherapy by upregulating the expression of the tumor antigen itself or MHC class I molecules that bind to the antigen to enhance tumor antigen presentation or enhance the intensity of effector T cell activity [19–21]. In contrast, chemotherapy can deplete lymphocytes, leading to immunosuppression. Another study showed that tumor mutational burden (TMB) was significantly reduced in NSCLC after chemotherapy, and the reduction of TMB was closely related to the response to immunotherapy [22]. In addition, the adverse events associated with long-term maintenance chemotherapy include hematological toxicity, abnormal liver and kidney function, fatigue, etc., which reduce the patient’s tolerance to treatment. These interactions may explain why pemetrexed in ICIs-based maintenance therapy among patients with PD-L1 TPS ≥1% did not get additional benefits.

The Checkmate-9LA study of 2-cycle chemotherapy plus two ICIs suggests that short-course chemotherapy can exert a rapid anti-tumor effect and avoid the adverse events caused by long-term chemotherapy [23]. A study showed that metronomic chemotherapy combined with ICIs is a promising treatment method, which can improve the activity of ICIs and maintain the effect of chemotherapy [24]. A commentary proposed the concept of “chemo-reform”: the addition of post-reform chemotherapy to immunotherapy, including single-drug chemotherapy (without platinum), platinum alone, low-dose chemotherapy, chemotherapy with the adjusted course, or cycle interval-adjusted chemotherapy [25]. But which mode is most beneficial to the patient is not known.

Our study has several limitations. First, the retrospective nature of our study may have influenced some outcomes, including the recording of adverse events. Second, the sample size of the ICIs monotherapy maintenance group in this study was small, mainly because the samples were from single-center. Third, the observation period is not long enough. Regarding OS, the number of events was immature. We would report it after follow-up. Finally, the discontinuation rate for pemetrexed in this study is noteworthy and may affect the generalizability of the results.

## Conclusions

In summary, our study showed that ICIs with or without pemetrexed can be used as maintenance therapy after first-line ICIs plus 4–6 cycles of pemetrexed/platinum in patients with advanced NS-NSCLC based on PD-L1 expression. In NS-NSCLC patients with PD-L1 TPS ≥1%, no statistically significant difference in efficacy was observed between ICIs monotherapy and ICIs plus pemetrexed as maintenance therapy; however, the efficacy of ICIs monotherapy maintenance was worse in patients with TPS < 1%. In the future, how to combine chemotherapy with immunotherapy to achieve optimal results and reduce adverse events warrants further reserch.

## Data Availability

The datasets used and analysed during the current study are available from the corresponding author on reasonable request.

## Abbreviations

NSCLC: Non-small cell lung cancer
NS-NSCLC: Non-squamous non-small cell lung cancer
ICIs: Immune checkpoint inhibitors
PFS: Progression-free survival
OS: Overall survival
NCCN: National Comprehensive Cancer Network
ECOG PS: Eastern Cooperative Oncology Group Performance Status
PD-L1: Programmed death ligand 1
CR: Complete response
PR: Partial response
SD: Stable disease
RECIST: Response Evaluation Criteria in Solid Tumors
MRI: Magnetic resonance imaging
ORR: Objective response rate
CTCAE: Common Terminology Criteria for Adverse Events
HR: Hazard ratio
95%CI: 95% confidence interval
TMB: Tumor mutational burden
